# A urinary microRNA panel that is an early predictive biomarker of delayed graft function following kidney transplantation

**DOI:** 10.1038/s41598-019-38642-3

**Published:** 2019-03-05

**Authors:** Usman Khalid, Lucy J. Newbury, Kate Simpson, Robert H. Jenkins, Timothy Bowen, Lucy Bates, Neil S. Sheerin, Rafael Chavez, Donald J. Fraser

**Affiliations:** 1 Wales Kidney Research Unit, School of Medicine, College of Biomedical & Life Sciences, Cardiff University, Heath Park Campus, Cardiff, CF14 4XN UK; 2 Institute of Cellular Medicine, Newcastle University, Newcastle upon Tyne, UK; 3 Newcastle upon Tyne Hospitals NHS Foundation Trust, Newcastle upon Tyne, UK; 4 Cardiff Transplant Unit, University Hospital of Wales, Cardiff, CF14 4XW UK

## Abstract

Predicting immediate and subsequent graft function is important in clinical decision-making around kidney transplantation, but is difficult using available approaches. Here we have evaluated urinary microRNAs as biomarkers in this context. Profiling of 377 microRNAs in the first urine passed post-transplantation identified 6 microRNAs, confirmed to be upregulated by RT-qPCR in an expanded cohort (miR-9, -10a, -21, -29a, -221, and -429, n = 33, P < 0.05 for each). Receiver operating characteristic analysis showed Area Under the Curve 0.94 for this panel. To establish whether this early signal was sustained, miR-21 was measured daily for 5 days post-transplant, and was consistently elevated in those developing Delayed Graft Function (n = 165 samples from 33 patients, p < 0.05). The biomarker panel was then evaluated in an independent cohort, sampled at varying times in the first week post-transplantation in a separate transplant center. When considered individually, all miRs in the panel showed a trend to increase or a significant increase in those developing delayed Graft Function (miR-9: P = 0.068, mIR-10a: P = 0.397, miR-21: P = 0.003, miR-29a: P = 0.019, miR-221: P = 0.1, and miR-429: P = 0.013, n = 47) with Area Under the Curve 0.75 for the panel. In conclusion, combined measurement of six microRNAs had predictive value for delayed graft function following kidney transplantation.

## Introduction

Kidney transplantation is the treatment of choice for many patients with end stage renal failure. It improves quality of life, prolongs survival and is more cost effective in comparison to dialysis^1,2^. Due to the increasing number of patients on the waiting list, new ways have been identified to increase the organ donor pool^3,4^. These have included the use of ‘marginal’ and ‘extended criteria’ donors (defined by the United Network for Organ Sharing (UNOS) as donors older than 60 years, or donors between age 50–59 with two or more criteria from higher terminal Creatinine >1.5 mg/dl, hypertension and death due to CVA)^5,6^. However, these innovations have also increased the impact of Ischemia Reperfusion Injury (IRI) on the kidney graft, increasing rates of delayed graft function (DGF, defined as the need for dialysis within the first week post-transplantation) and primary non-function. DGF increases length of hospital stay and risk of acute rejection episodes, and is associated with poorer long-term outcomes^7^.

Early ischemic injury, frequently manifesting as DGF, is also an important and potentially modifiable contributor to poor long term survival following kidney transplantation^8^. Risk of DGF may be predicted based on donor and recipient characteristics^9^. Graft function is intensively monitored post-transplantation, but separating ischemia-mediated DGF from other causes of graft dysfunction, and predicting extent and tempo of recovery of graft function, is difficult. Invasive biopsy of the kidney graft is frequently employed in order to differentiate DGF from other possible causes of graft dysfunction including acute rejection, and carries increased risk in the immediate post-transplant period^10^. Identifying a non-invasive biomarker of the extent of IRI and hence DGF, would improve prediction of outcome and tailored management for transplant patients. Development of such a biomarker would also facilitate testing of new approaches to attenuate IRI and reduce the risk of DGF.

Recently, microRNAs have emerged as essential post-transcriptional regulators of gene expression^11–13^. These small non-coding RNAs regulate various physiological and pathophysiological processes, and have an important role in acute kidney injury^14^. They are found in many biological samples including tissue, serum, and urine^15^. Because of their tissue-specific expression, microRNAs show promise as biomarkers, and their expression levels in urine may reflect intra-renal events^16^. Our laboratory has shown that microRNAs play a key role in renal proximal tubular cell recovery from acute kidney injury^17^ and has developed robust laboratory-based methodologies for the isolation and quantification of urinary microRNAs^16,18^.

The urinary microRNA profile shows promise for detection of acute rejection within the kidney graft^19–21^. Lorenzen *et al*. found that urinary miR-210 abundance was a sensitive discriminator for non-treated versus treated acute rejection^19^. A panel of urinary microRNA changes have also been reported in patients experiencing chronic allograft dysfunction^21^. However, to our knowledge no studies have evaluated the microRNA profile of DGF in kidney transplantation.

The aim of this study was to test whether by quantifying urinary microRNAs it is possible to predict those who will go on to develop DGF following kidney transplantation. Our approach was to perform unbiased profiling and confirmatory analysis in a discovery cohort from a single center. The resultant signature predictive of DGF was then validated in a second cohort of 47 patients transplanted at an independent center.

## Results

### Characteristics of the discovery cohort

Consecutive transplant recipients were recruited in a single transplant center at the time of transplantation, and with allocation to DGF or no DGF group at day 7 dependent on outcome. DGF was defined as requirement for Renal Replacement Therapy in the first 7 days post-transplantation^7^. To maximize capacity for results to translate into a test deliverable in clinical practice, total urinary microRNA quantification was performed, rather than sub-fractionation to study exosomes or other sub-compartments. To minimize sample variation in the profiling and discovery cohort, all patients were recruited in a single center, and the first urine passed post-transplantation was sampled from the urinary catheter immediately post-operatively. Pre-analytic sample handling and processing was adherent to the stated standard operating procedure, based on our previous work to define optimal handling and processing of urine samples for microRNA analysis.

HLA-incompatible transplantations were not included in the analysis, and all of the transplants included were from ABO-compatible donor-recipient pairs. Immunosuppression comprised Basiliximab or Anti Thymocyte Globulin and Methyl Prednisolone, followed by triple maintenance therapy (Tacrolimus, Mycophenolate Mofetil and Prednisolone). The same immunosuppressant protocol was employed for live and cadaveric recipients, however other differences in live versus cadaveric donor kidneys might influence post-transplantation urinary microRNA profile. In particular, the expected differences in pre-transplantation ischemic time were observed (Table 1) so in this initial analysis, live and cadaveric donor recipients were separated. Collection was continued until >10 patients were recruited to each of live-no DGF, cadaveric-no DGF, and cadaveric-DGF groups (these numbers based on expected magnitude of fold changes and variance, inferred from our previous urinary microRNA work). No live donor recipients sustained DGF during the collection period.

**Table 1:** Patient Demographics.

		Overall	Living donor without DGF	Cadaveric donor without DGF	Cadaveric donor with DGF
Donor median age in years (range)		48 (12–76)	43.5 (27–66)	54 (20–75)	65 (12–76)
Donor gender	Male	17	3	4	10
	Female	16	7	6	3
Donor cause of death	ICH	10	—	3	7
	HBI	2	—	2	0
	ICT	2	—	1	1
	Other	9	—	4	5
Median Cold Ischemic Time in minutes (range)		636 (46–1512)	192 (46–253)	870.5 (425–1512)	884 (456–1270)
Deceased Donor Type	DCD	12	—	3	9
	DBD	11	—	7	4
Recipient median age in years (range)		53 (18–76)	50.5 (18–61)	43 (19–74)	56 (39–76)
Recipient gender	Male	23	6	7	10
	Female	10	4	3	3
Dialysis Status	Pre-dialysis	5	5	0	0
	HD	18	3	5	10
	PD	10	2	5	3
Median Length of hospital stay in days (range)		8 (4–44)	6 (4–12)	7 (5–15)	19 (8–44)
Recipient cause of renal failure	DM	3	0	1	2
	IgA N	4	2	0	2
	GN	4			4
	APKD	6	4	1	1
	FSGS	5	2	2	1
	Other	12	3	6	3
Median no. of HLA mismatches (range)		4 (0–6)	4 (0–5)	4 (0–6)	4 (0–6)

**Figure 1:**
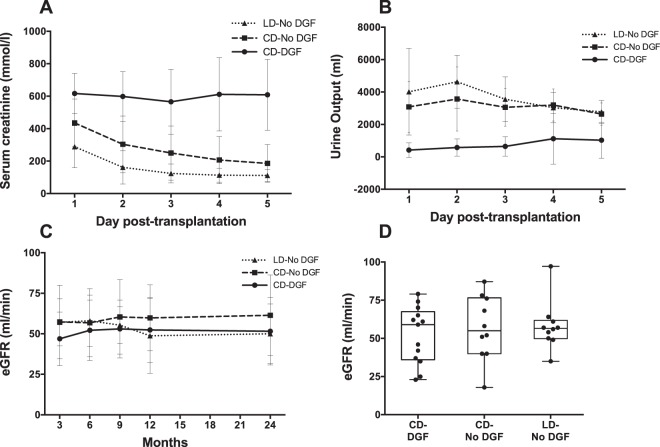
Serum Creatinine, Urine Output and estimated glomerular filtration rate post-transplantation. Consecutive kidney transplant patients were recruited into 3 groups: Living donor kidney transplant without DGF (LD-No DGF); Cadaveric donor kidney transplant without DGF (CD-No DGF); and Cadaveric donor kidney transplant with DGF (CD-DGF). The mean (±SD) serum creatinine (**A**) and urine output (**B**) were plotted for days 1 to 5 post-transplantation. The estimated glomerular filtration rate (eGFR) (in ml/min) was measured using the Modification of Diet in Renal Disease (MDRD) formula. Mean (±SD) eGFR was plotted at several time points post-transplantation (**C**) and eGFR (ml/min) at 6 months was also plotted (**D**). The boxes indicate first and third quartiles and the median, the whiskers indicate the maximum and minimum of the data, and individual values are shown as dots. Number of patients in each group: LD-No DGF (n = 10); CD-No DGF (n = 10); and CD-DGF (n = 13).

Clinical characteristics and demographics of the cohort are described in Table 1. 11 (48%) of deceased donor organs were from ‘Donation after brain-stem death’ (DBD) and 12 (52%) from ‘Donation after circulatory death’ (DCD) donors. When comparing those developing versus not developing DGF, similar numbers of donors had as cause of death Intracerebral hemorrhage (ICH), Hypoxic Brain Injury (HBI), Intracerebral Thrombosis (ICT), and other causes. Recipient cause of renal failure demonstrated similar numbers of recipients with Diabetic Kidney Disease (DM), Glomerulonephritis (GN), Adult Polycystic Kidney Disease (APKD) and Focal Segmental Glomerulosclerosis (FSGS). Patients experiencing DGF had longer hospital stays (median 19 days) compared to cadaveric kidney recipients not developing DGF (7 days) or live donor recipients (6 days). Two patients moved out of the area after 6 months. Duration of follow up was 2 years for all the other patients. Three episodes of graft loss were recorded (1 at 3 months (declining kidney function); 2 at 13 and 20 months respectively (rejection)). No patient mortality was recorded. Eight (24%) patients experienced one or more acute rejection episodes. Rejection rates were 31%, 10%, and 30% in the ‘CD-DGF’, ‘CD-No DGF’, and ‘LD-No DGF’ groups respectively (p = 0.452). Five of eight rejection episodes were “borderline” or “suspicious” for rejection according to Banff ’97 Working Classification^22^. The other three led to graft loss, and comprised one CD-DGF loss at three months due to further deterioration in a recipient with poor post-transplant baseline function, and two LD-No DGF losses (at 13 and 20 months) due to severe rejection, in both cases there was possible noncompliance with immunosuppressive medication. In all cases, biopsies followed a clinical suspicion of rejection, and no protocol biopsies were undertaken in patients in this cohort. Mean estimated glomerular filtration rate (eGFR) (±SEM) at 6 months post-transplantation was 55.33 (±3.17) ml/min and there was no significant difference in eGFR between the 3 groups (p > 0.9) (Fig. 1).

### Identification of microRNAs differentially expressed in urine from patients subsequently developing DGF

**Figure 2:**
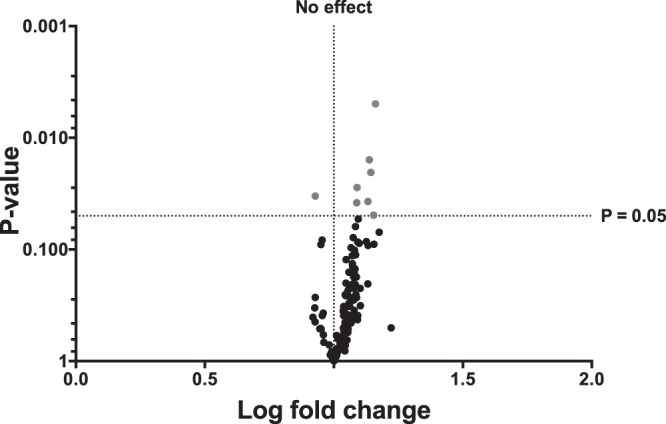
Volcano plot of 137 microRNAs detected in ‘first-pass’ urine from cadaveric donors subsequently developing versus not developing delayed graft function (DGF). Taqman Low Density Array (TLDA) quantification was performed to profile the expression of 377 microRNAs in urine collected 24 h post-transplantation in a “discovery set” comprising ‘first-pass’ urine of 4 recipients of ‘living-donor kidney transplant without DGF’ (LD-No DGF) compared to 4 recipients of ‘cadaveric kidney transplant with DGF’ (CD-DGF). Independent profiling was performed on each urine sample before normalisation to miR-cel-39. Relative expression was calculated by the delta delta Ct method^42^. A volcano plot of statistical significance (p-value) against fold-change was plotted between ‘LD-No DGF’ and ‘CD-DGF’. The most differentially expressed microRNAs were selected as candidates for further analysis (miR-9, -10a, -21, -29a, -221, -429, -506, and -574-3p).

Initial microRNA profiling was performed, to identify candidate microRNAs that might signal underlying graft ischemic injury and hence risk of DGF. 377 microRNAs were profiled by Taqman Low Density Array in four live donor recipients with immediate kidney function and four patients with cadaveric donor kidneys who went on to develop DGF. There was an overall pattern of increased abundance of microRNAs in the samples from those subsequently developing DGF (Fig. 2).

#### Validation of target microRNAs

**Figure 3:**
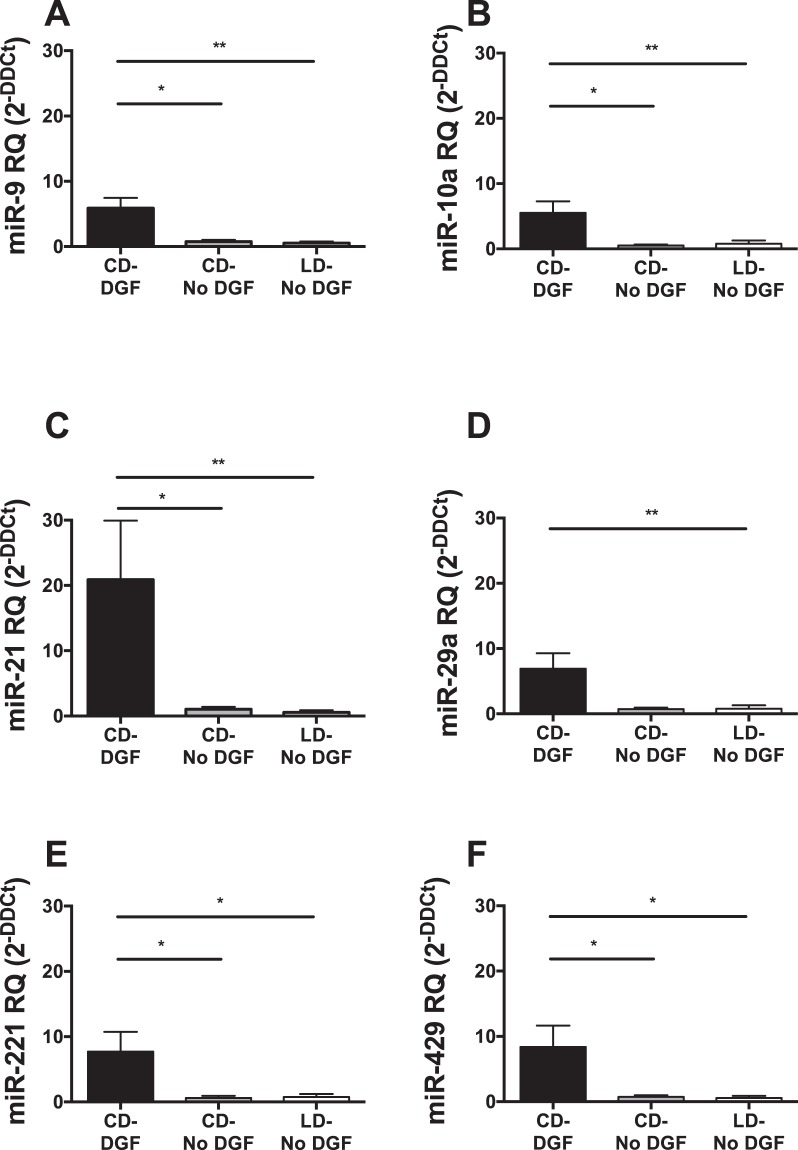
RT-qPCR analysis of microRNAs differentially expressed in DGF. MicroRNAs identified as differentially expressed in TLDA profiling (Fig. 2) were quantified by RT-qPCR in ‘first-pass’ urine in 33 patients, whose donor and recipient characteristics are described in Table 1. MicroRNAs were quantified by Taqman individual microRNA assay, and data are normalised to miR-Cel-39 before calculation of relative expression by the delta delta Ct method^42^. 6 of 8 target microRNAs were up regulated in CD-DGF. Data are plotted as mean ± SEM. Number of patients in each group: LD-No DGF (n = 10); CD-No DGF (n = 10); and CD-DGF (n = 13). Statistical significance: *p < 0.05, **p < 0.01.

**Figure 4:**
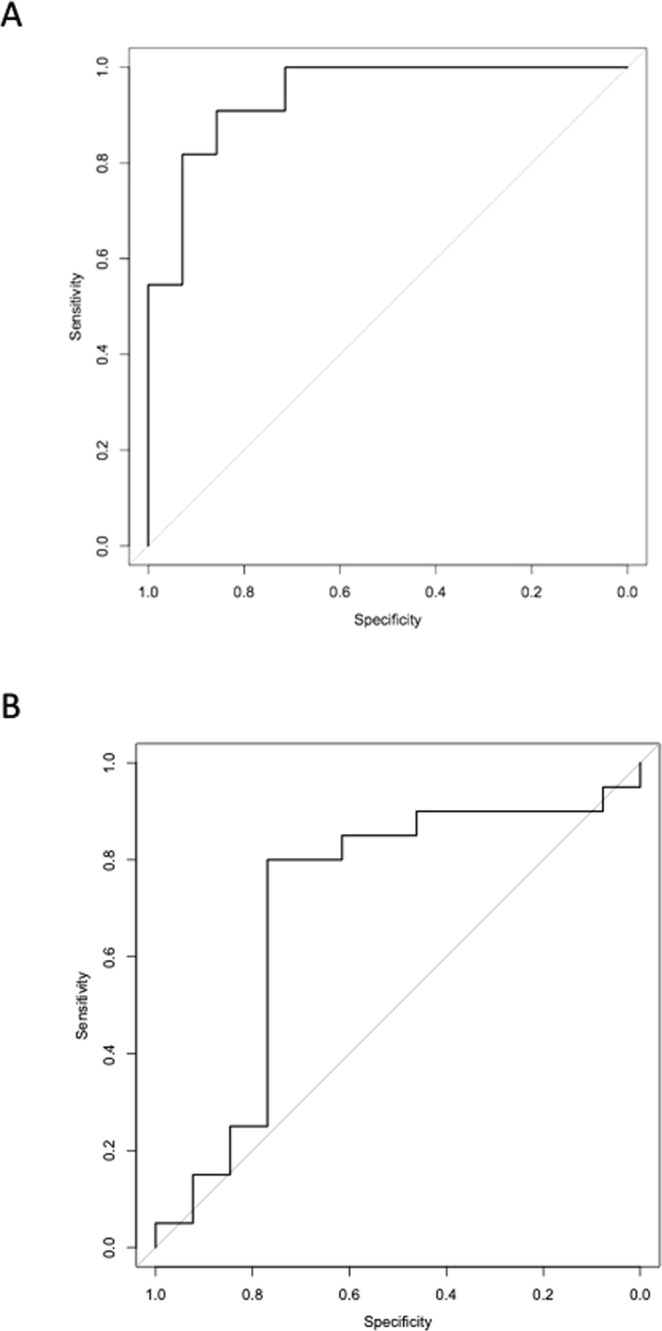
Receiver Operating Characteristic (ROC) curve analyses of 6 microRNA signature to discriminate between patients with DGF and those without DGF. ROC Curve Analysis, plotting sensitivity versus specificity for the Cardiff Cohort of patients developing DGF versus No DGF. Performance of discovered microRNA signature and of published predictive algorithm based on donor and recipient characteristics are presented. (**A**) ROC Curve for 6-microRNA signature (miR-9, -10a, -21, -29a, -221, and -429), Area under ROC 0.94. (**B**) ROC Curve for the 6 microRNAs which were up-regulated in DGF.

From the TLDA profiling quantification, 8 microRNAs, which were most significantly differentially expressed in ‘CD-DGF’ group compared with ‘LD-No DGF’ group, were chosen for confirmatory RT-qPCR. Expression of 6 microRNAs (miR-9, -10a, -21, 29a, -221, and -429) was significantly up-regulated with ≥5-fold change in the ‘CD-DGF’ group compared with both ‘LD-No DGF’ and ‘CD-No DGF’ groups, at ‘first-pass urine’ post-transplantation (Fig. 3). Expression of miR-574-3p and -506 was not significantly differentially expressed. No differences were detected between live and cadaveric donor recipients (Fig. 3) so further analysis focused on DGF occurrence independent of donor type. Combined Receiver Operating Characteristic (ROC) curve analysis of the 6 microRNAs (miR-9, -10a, -21, 29a, -221, and -429) showed ROC-AUC of 0.94 for discrimination of DGF from no-DGF outcome (Fig. 4). This was superior to the results obtained for any individual miR (miR-9: AUC = 0.90, miR-10a: AUC = 0.82, miR-21: AUC = 0.81, miR-29a: AUC = 0.79, miR-221: AUC = 0.80, miR-429: AUC = 0.79) suggesting that the optimal approach was measurement of the combined biomarker panel. In comparison, ROC Curve analysis of this dataset employing the predictive scoring system of Irish *et al*.^23^ based on donor- and recipient-clinical characteristics, gave ROC-AUC 0.71 (Fig. 4).

### Urinary expression profile of miR-21 in the first five days post-transplantation

**Figure 5:**
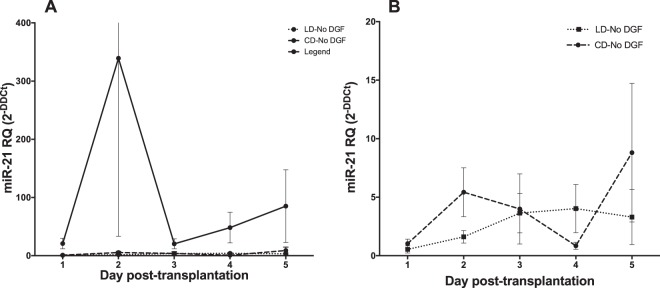
The urinary expression profile of miR-21 post-transplantation. MiR-21 was one of 6 microRNAs up-regulated in ‘first-pass’ urine samples of kidney transplant patients with DGF (Fig. 3). RT-qPCR was performed, using Taqman individual microRNA assay, for miR-21 to determine its expression profile in the first 5 days post-transplantation. Data was normalised to miR-Cel-39 before calculation of relative expression by the delta delta Ct method^42^. Data are plotted as mean ± SEM. Number of patients in each group: LD-No DGF (n = 10); CD-No DGF (n = 10); and CD-DGF (n = 13).

The above data identified a panel of microRNAs that were predictive of DGF when measured in urine collected immediately post-transplantation. In order to determine whether urinary microRNAs are more generally useful as a biomarker of DGF in clinical practice, we next evaluated urinary expression profile of miR-21 in this cohort for the 5 consecutive days after kidney transplantation. miR-21 in urine was significantly up-regulated in the ‘CD-DGF’ group compared with both the ‘No DGF’ groups during the first five days post-transplantation (p < 0.05) (Fig. 5). When the ‘CD-No DGF’ and ‘LD-No DGF’ groups were compared, no significant difference in miR-21 expression levels was found. These data suggest that the increased microRNA expression in urine of patients with DGF identified here is sustained in the days following transplantation.

### Independent Validation of results using a second cohort

**Figure 6:**
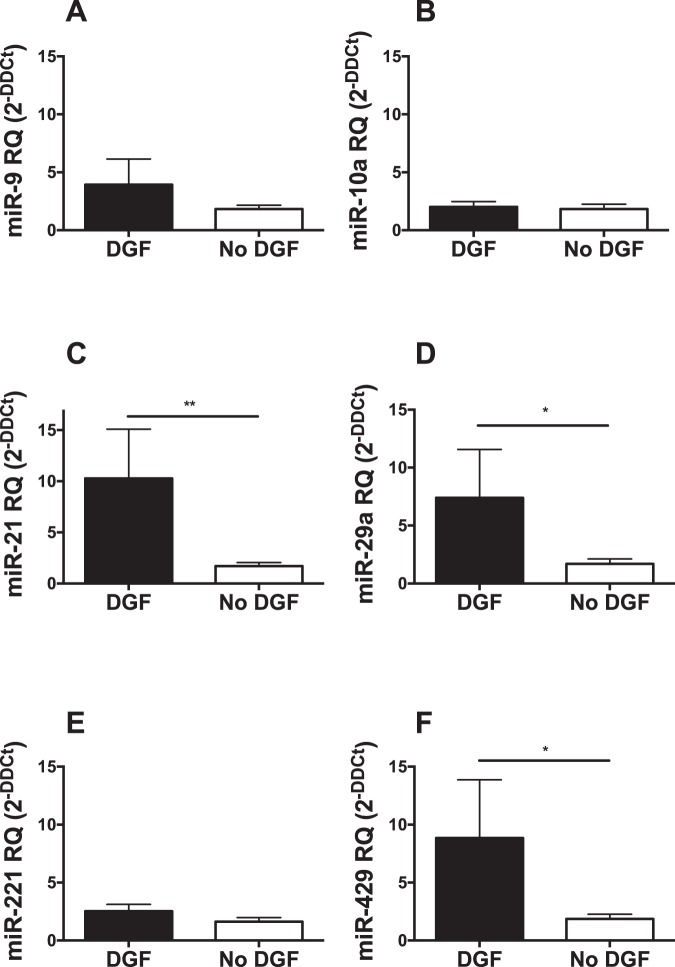
RT-qPCR analysis of microRNAs differentially expressed in DGF in a second validation cohort from a different transplant centre. Urine samples were collected in the first week post-transplantation from 50 kidney transplant recipients at Newcastle transplant centre and grouped into ‘DGF’ (n = 13) and ‘No DGF’ (n = 37). Following RNA extraction, RT-qPCR was performed to quantify the 6 validated microRNAs (miR-9, -10a, -21, -29a, -221, and -429) using Taqman individual microRNA assays. Data was normalised to miR-191 before calculation of relative expression by the delta delta Ct method^42^. Data are plotted as mean ± SEM.

**Figure 7:**
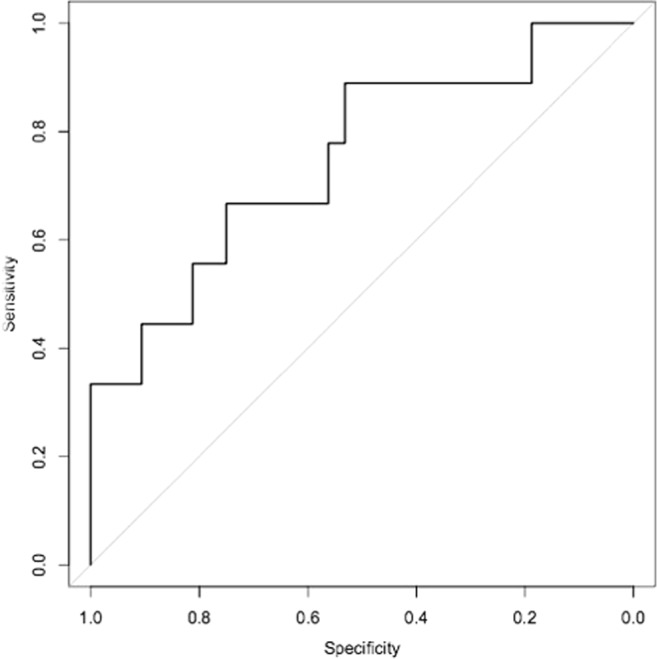
Receiver Operating Characteristic (ROC) curve analyses of 6 microRNA signature to discriminate between patients with DGF and those without DGF. Newcastle cohort: DGF (n = 13) v No DGF (n = 37) ROC of 0.75 for the 6 microRNAs which were up-regulated in DGF (miR-9, -10a, -21, -29a, -221, and -429).

In order to test the biomarker panel and to validate these findings, an independent cohort of 47 patients transplanted at a different transplant center (Newcastle Institute of Transplantation Tissue Biobank) was recruited and their urine samples collected in the first week post-transplantation. Of the 47 patients recruited, 13 (28%) developed DGF and 34 (72%) had no DGF (demographic data is displayed in Table 2). Mean estimated glomerular filtration rate (eGFR) (±SEM) at 6 months post-transplantation was 48.19 (±2.71) ml/min and no significant difference in eGFR was detected between the 2 groups (p = 0.089). In order to provide a robust analysis of the panel in clinical practice, samples were collected throughout the first week post-transplantation. This led to increased variability of individual microRNA measurements (Fig. 6) consistent with the pattern observed in daily measurement of miR-21 (Fig. 5). All microRNAs showed a trend to- or significantly increased-expression in patients developing DGF (Fig. 6). A combined ROC curve analysis of the panel gave a ROC-AUC of 0.75 (Fig. 7).

**Table 2:** Patient Demographics of validation cohort (Newcastle).

		Overall (n = 47)	DGF (n = 13)	No DGF (n = 34)
Donor median age in years (range)		54.5 (28–75)	56 (40–74)	52 (28–75)
Donor gender	Male	29	9	20
	Female	18	4	14
Donor cause of death (n = 30)	ICH	17	8	9
	HBI	7	1	6
	ICT	1	1	0
	Other	5	2	3
Median Cold Ischemic Time in minutes (range)		717.5 (45–1495)	777 (74–1148)	628 (45–1495)
Deceased Donor Type (n = 30)	DCD	18	9	9
	DBD	12	3	9
Recipient median age in years (range)		51 (18–72)	54 (38–66)	51 (18–72)
Recipient gender	Male	33	10	23
	Female	14	3	11
Dialysis Status	Pre-dialysis	15	2	13
	HD	26	8	18
	PD	6	3	3
Median Length of hospital stay in days (range)		15 (9–63)	18 (12–63)	13 (9–38)
Recipient cause of renal failure	DM	3	1	2
	IgA N	6	2	4
	GN	7	1	6
	APKD	5	1	4
	FSGS	1	1	0
	Other	25	7	18
Median no. of HLA mismatches (range)		3 (0–6)	3 (0–4)	3 (0–6)

## Discussion

Here, we have used unbiased profiling to identify microRNAs in the urine that are predictive of DGF following kidney transplantation (miR-9, -10a, -21, -29a, -221, and -429) and subsequently confirmed these findings using measurement of specific microRNAs by RTqPCR. These alterations in miR detection were then tested in an independent cohort of patients transplanted at a separate center, and were further shown to be evident through the five days following transplantation. These data suggest that urinary miR profile may be a useful measure of renal injury that manifests subsequently as delayed graft function.

Recent studies have begun to uncover the likely functions of microRNAs in the kidney, and there is accumulating evidence that microRNAs play a key role in responses to acute kidney injury of various etiologies^20,24,25^. Amongst these, the most is known in terms of kidney injury about the role of miR-21. Increased levels of miR-21 have been found in the urine and plasma of patients sustaining AKI of various etiologies^26–28^, and abundant data demonstrates mechanistic importance of miR-21 in the kidney following ischemic injury, where miR-21 may serve both to limit acute injury by inhibition of apoptosis and inflammatory pathways but also in the longer term may promote fibrosis (reviewed in^29^). In addition, our recent work showed that miR-21 measured in hypothermic machine perfusate of kidneys placed on the Lifeport® perfusion system correlated with estimated GFR at 6 and 12 months post-transplantation^30^. In the current study, consistent with its possible role as a sentinel for ischemic injury in the donor kidney, we found that elevated miR-21 was detectable throughout the first five days post-transplantation in patients developing DGF. Importantly, however, miR-21 as a single biomarker was outperformed by a combined analysis of the signature derived from unbiased profiling.

The functions of other microRNAs identified in the present study in the context of kidney transplantation are currently unclear. Many of the known roles of these microRNAs are in cancer and development, potentially reflecting shared underlying actions but also potentially affected by the critical mass of research focus and studies in the field to date. MiR-10a is abundantly expressed in normal mouse kidney, where its function is unknown. It targets Hox genes, transcription factors with key developmental roles, and is found at increased levels in diverse tumors (reviewed in^31^). Previous data supports miR-10a as a biomarker in mouse models of AKI^32^. MiR-29a is abundant in kidney, where it is linked to matrix deposition in response to the profibrotic cytokine Transforming Growth Factor Beta-1^33^ as well as, outside the kidney, to regulation of the immune system and tumorigenesis (reviewed in^34^). MiR-221 regulates angiogenesis and is highly expressed in multiple cancers, where it has been proposed as both a biomarker and therapeutic target^35^. MiR-429 is part of the miR-200 family, strongly implicated in retention of an epithelial phenotype and downregulated in cancer-associated epithelial to mesenchymal transition^36^ while MiR-9 is implicated in brain development^37^.

Previous studies have evaluated microRNAs as potential biomarkers in kidney transplant recipients, focusing to date on detecting acute rejection^19,38^. Anglicheau *et al*. found increased amounts of miR-142-5p, -155 and -223 in kidney biopsies from 12 patients experiencing acute rejection, and linked this to infiltration of the allograft by multiple cell types^38^. Lorenzen *et al*. found decreased levels of miR-210 in the urine of 62 patients suffering an acute rejection episode, and that low miR-210 levels were associated with a greater rate of decline in excretory kidney function in the first year^19^. These microRNAs were not differentially expressed in our profiling studies, supporting the idea that different microRNA changes are seen in patients exhibiting DGF versus those suffering an acute rejection episode, consistent with the different underlying pathology.

ROC analysis is a commonly used approach to depict the trade-offs between sensitivity and specificity for a given test. The ROC curve can be summarized by the area under the curve (AUC ROC) which equates to the probability that if a random case and control from the population are compared, the case will have the higher value (reviewed in^39^). The microRNA panel that we describe had very high discriminatory capacity in the discovery set (AUC 0.94) which was reduced in a second, independent validation cohort (AUC 0.75). In part, this reflects the inherently higher predictive value for a biomarker panel in the sample set from which it is defined. Important concepts for validation are replication in an independent cohort, and to seek clinical as well as statistical validation (reviewed in^40^). In the current study, the validation cohort was chosen to reflect how the test might be used in standard care, with sampling at variable times in the immediate post-transplant period. A consequence is greater variability in microRNA levels, as reflected in Fig. 5, and we propose that this diminishes the performance of the test (in AUC terms) but gives a more realistic appreciation of its potential value in clinical practise.

DGF Scoring systems using clinical parameters have previously been developed, for example that by Irish *et al*. which uses 20 donor- and recipient-variables to predict likelihood of DGF^23^. The AUC for this predictive tool was reported as 0.704 when first described, and consistent with this, we found an AUC ROC of 0.709 employing this approach. The AUC for the urinary microRNA profile that we have identified performs well when compared to this, with AUC 0.75 in the external validation cohort. Rather than seeking to replace consideration of existing clinical parameters, however, optimal use of the profile that we have identified is likely to involve its integration with such data. Such a combined approach has the potential advantage of integrating multiple data points about an individual patient, each of which may inform about different components of their overall condition.

MicroRNAs are emerging as important biomarkers in multiple disease processes including kidney injury and transplantation. The current study identifies a microRNA signature in urine that provides a non-invasive measure of DGF risk in kidney transplant recipients. It will be important in future studies to test the utility of this measure in the decision-making processes surrounding kidney transplantation, and to determine how best to integrate it with prediction approaches based on baseline characteristics and other clinically available data. It will also be useful to seek to understand the mechanisms underlying the increased urinary miR levels that we have uncovered. In the case of miR-21 there is strong evidence to link this to link this to its known regulatory actions within kidney cells, in the case of the other miRs identified, the underlying mechanisms are to be discovered, and represent an important future focus for research.

## Methods

All methods were carried out in accordance with relevant guidelines and regulations. Experimental protocols were approved by Cardiff University, and informed consent was obtained from all subjects.

### Urine Samples

Urine samples were collected in 20 ml universal containers from consecutive kidney transplant patients on days 1 to 5 post-transplant at a single transplant center (Cardiff Transplant Center, University Hospital of Wales, Cardiff, UK). The first sample was taken immediately post-transplant, i.e. ‘first-pass urine’. Recruitment took place until there were at least 10 patients per group: living donor kidney transplant without DGF (LD-No DGF), cadaveric donor kidney transplant without DGF (CD-No DGF), and cadaveric donor kidney transplant with DGF (CD-DGF). All patients gave informed consent for samples to be collected and stored in the Wales Kidney Research Tissue Bank. Urine samples were centrifuged at 2000 g for 10 minutes at 4 °C to remove debris. The supernatant was divided into aliquots of 350 μl for RNA extraction. Samples were stored at −80 °C until RNA extraction experiments. For subsequent validation, a second cohort of 47 patients undergoing transplantation at a second center (Newcastle Institute of Transplantation) was recruited. Patients in this validation cohort gave a single spot sample of urine in the first five days following transplantation. All subjects gave informed consent for sample collection (Research Ethics Committee reference 11/NE/0352 and 09/WSE02/48).

### RNA Extraction

RNA was extracted using miRNeasy Mini Kits (Qiagen, Manchester, UK) as per the manufacturer’s instructions, with minor modifications as per the protocol recently established in this laboratory^41^. In brief, urine was centrifuged at 2000 *g* for 10 mins at 4 °C before addition of carrier RNA (MS2 RNA, Roche) at 1 μg/750 μL w/v, and synthetic Caenorhabiditis elegans cel-miR-39 (MC10956, cel-miR-39; Life Technologies, Paisley, Renfrewshire, UK) to final concentration 0.5pM, before proceeding as per the miRNeasy extraction protocol.

### Taqman Low Density Array

377 unique microRNAs and 4 controls were measured by Taqman Low Density Array (TLDA, Megaplex RT Primers Human Pool A v.2.1) with Reverse Transcription followed by pre-amplification Megaplex PreAmp primers, according to the manufacturer’s recommendations (Life technologies). TLDA (Human MicroRNA Panel Card A v.2.0) was performed using a 7900-HT Fast Real-Time PCR System, according to the manufacturer’s recommendations (Life Technologies).

### RT-qPCR

cDNA was generated using a High Capacity Reverse Transcription Kit with specific stem loop primers for the TaqMan miRNA assays (Life Technologies). RT-qPCR was performed on a ViiA7 Fast Real-Time PCR System (Life Technologies). The amplification of a single PCR product was confirmed by melt curve analysis. Selected microRNAs (miR-9, -10a, -21, -29a, -221, -429, -506, and -574-3p) were quantified by Taqman miRNA assay, according to the manufacturer’s instructions and their expression normalized to miR-cel-39. The relative changes in gene expression were analyzed by the 2^−ΔΔCT^ method^42^. Taqman microRNA gene expression assay ID numbers were hsa-miR-9 (000583), hsa-miR-10a (000387), hsa-miR-21 (000397), hsa-miR-29a (000412), hsa-miR-221 (000524), hsa-miR-429 (001024), hsa-miR-506 (001050), hsa-miR-574-3p (002349) and miR-cel-39 (000200).

### Clinical Data

The clinical and research activities being reported are consistent with the Principles of the Declaration of Istanbul as outlined in the ‘Declaration of Istanbul on Organ Trafficking and Transplant Tourism’. Demographic data were collected on the donors (age, gender, cause of death for deceased donors, cold ischemic times) and recipients (age, gender, cause of renal failure, dialysis status,), HLA mismatch, duration of hospital stay, and eGFR at 3, 6, 9 and 12 months.

### Statistics

Statistical analyses were performed using GraphPad Prism version 6. ROC analysis was conducted using R software. Categorical Data were expressed as median (and range) and analyzed by Mann-Whitney U test. Continuous data were reported as mean (±SEM) and analyzed by unpaired Student’s *t* test. Significance level was pre-specified as p-value < 0.05.
